# Historical H1N1 Influenza Virus Imprinting Increases Vaccine Protection by Influencing the Activity and Sustained Production of Antibodies Elicited at Vaccination in Ferrets

**DOI:** 10.3390/vaccines7040133

**Published:** 2019-09-28

**Authors:** Magen E. Francis, Mara McNeil, Nicholas J. Dawe, Mary K. Foley, Morgan L. King, Ted M. Ross, Alyson A. Kelvin

**Affiliations:** 1 Department of Microbiology and Immunology, Faculty of Medicine, Dalhousie University, Halifax, NS B3H 4R2, Canada; m.francis@dal.ca (M.E.F.); mara.mcneil@dal.ca (M.M.); nc350339@dal.ca (N.J.D.); mr471027@dal.ca (M.K.F.); morg.king@icloud.com (M.L.K.); 2 Center for Vaccines and Immunology, Department of Infectious Diseases, University of Georgia, Athens, GA 30602, USA; tedross@uga.edu; 3 Department of Pediatrics, Division of Infectious Disease, Faculty of Medicine, Dalhousie University, Halifax, NS B3K 6R8, Canada; 4 Canadian Centre for Vaccinology, IWK Health Centre, Halifax, NS B3K 6R8, Canada

**Keywords:** influenza virus, imprinting, Haemagglutinin, antibody titer, quadrivalent vaccine, influenza A, H1N1, split-virion, isotype, virus neutralization

## Abstract

Influenza virus imprinting is now understood to significantly influence the immune responses and clinical outcome of influenza virus infections that occur later in life. Due to the yearly cycling of influenza viruses, humans are imprinted with the circulating virus of their birth year and subsequently build a complex influenza virus immune history. Despite this knowledge, little is known about how the imprinting strain influences vaccine responses. To investigate the immune responses of the imprinted host to split-virion vaccination, we imprinted ferrets with a sublethal dose of the historical seasonal H1N1 strain A/USSR/90/1977. After a +60-day recovery period to build immune memory, ferrets were immunized and then challenged on Day 123. Antibody specificity and recall were investigated throughout the time course. At challenge, the imprinted vaccinated ferrets did not experience significant disease, while naïve-vaccinated ferrets had significant weight loss. Haemagglutination inhibition assays showed that imprinted ferrets had a more robust antibody response post vaccination and increased virus neutralization activity. Imprinted-vaccinated animals had increased virus-specific IgG antibodies compared to the other experimental groups, suggesting B-cell maturity and plasticity at vaccination. These results should be considered when designing the next generation of influenza vaccines.

## 1. Introduction

Influenza A virus infection is a persistent challenge to public health. Two types of Influenza viruses (Orthomyxoviridae), influenza A and influenza B viruses, each with its own subtypes and lineages, currently circulate in humans [1,2,3]. These viruses cause millions of hospitalizations and thousands of deaths yearly infecting between 5% and 30% of the global population [4,5,6,7,8]. Furthermore, infants, the elderly, pregnant women, and people with pre-existing medical conditions are at higher risk of developing severe disease requiring hospitalization from influenza infection [9].

The well-known yearly cycling of the influenza virus is the result of the reciprocal relationship between the host and the virus: human immune responses influence virus mutation which feeds back to influence immune response [8]. As a result, vaccines need to be reformulated every year, at great expense, to match circulating strains [10]. The changes in the influenza virus occur through a process known as antigenic drift [11]. Due to antigenic drift, distinct seasonal strains emerge each year with the ability to infect a vulnerable human population [12]. The very first influenza virus exposure in a human during infancy is known to imprint the host immune system, leading to virus-specific B-cell and T-cell memory clones that are long-lived and skewed toward antigenic sites of the imprinting virus [13]. For the humoral arm of the immune system, imprinting leads to antibodies and memory B cells that first target virus epitopes which remain throughout life and may impact future infections [14]. Over time, humans build a complex immune history as they are cyclically infected with novel influenza strains and receive seasonal vaccinations [15,16,17]. It is recognized that the immune responses from the imprinting infection which leads to *preimmunity* (the entire host influenza history) significantly influences vaccine and infection outcomes, but the mechanisms that regulate vaccine responses in the imprinted/preimmune host have yet to be elucidated [13,18,19,20].

The host’s primary infection with an influenza virus initiates a cascade of innate and adaptive immune events that culminate in immunological memory. This first infection in a person’s lifetime is referred to as the viral imprinting event [14]. The goal of the immune response is to mobilize adaptive immunity for the production of antibodies capable of virus neutralization. This response is defined by the generation of short-lived plasma cells (antibody producing cells) [21]. Activated B cells mature into plasmablasts and plasma cells, producing antibodies targeted at viral epitopes. The adaptive cellular and humoral responses move through three phases: expansion, contraction, and memory. The expansion phase is the proliferation of antigen-specific T and B cells producing a large number of reactive cells to control the pathogen. Experimental studies from our group, as well as others investigating immune responses in animal models (non-human primate and ferret), have shown that lymphocytes as well as virus-specific antibodies circulate in high numbers (in the expansion phase) even after pathogen clearance (up to 48 days) [22,23]. Eventually, adaptive immune cells contract until there is a limited number of highly specific cells able to circulate for immune surveillance, which is referred to as the B cell memory pool [24]. Considering the seasonality of influenza virus infection and vaccination, the memory phase would be the time period in which humans are typically re-exposed.

Immune history has an impact on host influenza vaccination responses [14,25,26]. Human studies based on serology or epidemiological study designs have suggested that understanding how the host interacts with an antigenically divergent pathogen, such as influenza viruses over multiple exposures will be important for identifying susceptible populations and designing the next generation of influenza vaccines. Here, we investigated the responses to the split, inactivated virion Sanofi QIV (Quadrivalent Influenza Vaccine) influenza virus vaccine (Sanofi Pasteur, North York, ON, Canada) in ferrets that had been imprinted with the historical H1N1 virus strain A/USSR/90/1977 (USSR/77). We first established preimmunity over 67 days to ensure that the ferrets were outside of the expansion phase of the adaptive immune response. This was done to avoid non-specific immunological interference lingering from the initial infection. Vaccinated ferrets that were previously infected with the historical seasonal influenza virus strain A/USSR/90/1977 had significantly divergent responses in terms of antibody profiles and clinical disease compared to naïve-vaccinated ferrets. These results have implications on future vaccine design and evaluation.

## 2. Materials and Methods

### 2.1. Ethics Statement

All animal research was conducted in strict accordance with the Canadian Council of Animal Care (CCAC) guidelines. The protocol license numbers AUP 5316 and 1031 were assigned by the Animal Care Committee of the University Health Network (UHN). UHN has certification with the Animals for Research Act, including for the Ontario Ministry of Agriculture, Food and Rural Affairs, Permit Numbers: #0132–01 and #0132–05, and follows NIH guidelines (OLAW #A5408-01). The animal use protocol was approved by the UHN Animal Care Committee (ACC). All efforts were made to minimize animal suffering. Infections and sample collections were performed under 5% isoflurane anesthesia.

### 2.2. Influenza Virus and Animals

The 2009 H1N1 virus strains A/California/07/2009 (Cal/07), A/USSR/90/1977 (USSR/77), and A/Taiwan/1/1986 (Taiwan/86) were provided by the Influenza Reagent Resource, Influenza Division, WHO Collaborating Center for Surveillance, Epidemiology and Control of Influenza, Centers for Disease Control and Prevention, Atlanta, GA, USA. TCID_50_ and EID_50_ determinations were done as previously described [27]. All virus work was performed in a BSL2+ facility as previously described [28]. Sanofi FLUZONE^®^ quadrivalent QIV influenza vaccine from the 2015–2016 influenza season was acquired from Sanofi Canada (North York, ON, Canada). The vaccine contained concentrated HA proteins from A/California/07/2009 (H1N1); A/Victoria/210/2009 (H3N2); B/Brisbane/60/2008 (B-Victoria Lineage); B/Florida/04/2006 (B-Yamagata Lineage). Adult female ferrets (aged ~5 months to 1 year) were purchased from Triple F Farms (Gillett, PA, USA). Ferrets were determined to be seronegative by haemagglutination inhibition (HAI) assay against currently circulating influenza A and B strains before infection. 

### 2.3. Infections and Vaccinations

Ferret were infected intranasally as previously done [29]. Briefly, ferrets were anesthetized and infected with seasonal viruses or pandemic viruses at 10^6^ EID_50_. The volume of inoculum was 1 mL for each ferret (0.5 mL in each nare). For vaccination, ferrets were vaccinated intramuscularly with a whole human dose of FLUZONE^®^ Sanofi QIV vaccine in the upper hind limb. Ferrets were observed post infection or vaccination for adverse effects.

### 2.4. Clinical Monitoring

Weight, temperature, and clinical signs were monitored following infections and vaccinations for 14 days similarly as previously done [30]. Weight and temperature for each day were calculated as a percentage of original values determined on Day 0 and days prior to study initiation. Standard deviation and standard error were calculated for weight and temperature percentages within each group. Clinical signs (body temperature, body weight, level of activity, nasal discharge, and sneezing) were observed daily for 14 days post imprinting/infection (*p*i), post vaccination, or post challenge (*p*c). Ferrets were examined at the same time each day for consistency. Nasal discharge, sneezing, and inactivity was observed and recorded but not reported here.

### 2.5. Viral Titers, HAI (haemagglutination inhibition) Titers, Microneutralization Titers (MN), and IgG/IgM Relative Isotype Levels

Viral titers were calculated from collected nasal wash samples *p*c. Nasal washes were subjected to a TCID50 assay followed by an HA (haemagglutination) assay against the virus strain of interest to determine viral titers. Viral load was calculated using the Reed and Muensch method [31].

HAI titers were determined in serum collected throughout the entire study by HAI assay using USSR/77, Cal/09, or Taiwan/86 live viruses or using the WHO circulating influenza virus detection kit (2016–2017). The WHO kit contains antigen for H1N1 2009, circulating H3N2, circulating B-Yamagata, and circulating B-Victoria strains. HAI assays were conducted as previously described [31]. Briefly, serum samples were treated with receptor-destroying enzyme (RDE) at 37 °C overnight. Fresh turkey red blood cells (TRBC) were prepared at a concentration of 0.5% (vol/vol) in PBS after washing. The sera were serially diluted in PBS in 96-well V-bottom cell culture plates and incubated with 25 μL (8 HA U/50 μL) virus for 15 min. Then, 50 μL of 0.5% TRBC was added, and the plates were incubated at room temperature for 30 min. The haemagglutination inhibition (HAI) titer was determined as the reciprocal of the highest serum dilution to completely prevent agglutination. HAI Units were calculated by the Log2 of the denominator of the HAI titer as done previously [32,33].

MN titers were evaluated by enzyme-linked immunosorbent assay (ELISA). Neutralizing antibody titers were determined by the highest dilution of RDE-treated anti-sera that disrupted infection (100 TCID_50_) on MDCK cells at the reading lower than 50% signal reading measured from virus + cell and cell only controls [31].

The determination of IgG and IgM specific antibodies in collected serum was based on enzyme-linked immunosorbent assay (ELISA) technique as previously described [34]. Briefly, ELISA plates were directly coated with A/California/07/2009 overnight at room temperature. Plates were washed with PBS containing 0.05% Tween 20 (T-PBS) and blocked with 1% bovine serum albumin (BSA) for 1 h at 37 °C. Antigen-coated plates were incubated with 1:1000-diluted serum samples overnight at 4 °C. After washing with T-PBS, plates were incubated with goat anti-ferret immunoglobulin (IgM and IgG) horseradish peroxidase (HRP) conjugates (Rockland Immunochemicals) in a 1:10,000 dilution for 2 h at 37 °C. The reaction was developed by *o*-phenylenediamine for 30 min, and the optical density was read at 492 nm.

### 2.6. Histopathology

Lungs were collected at necropsy, perfused with formalin and paraffin embedded. Following sectioning, the tissues were mounted and H&E stained. High resolution scans were performed using an Aperio ScanScope XT (Leica Biosystems, Concord, Canada) at 40× magnification. Images were captured using the the HALO program from UHN AOMF (Advanced Optical Microscopy Facility) at 5×, 10×, or 20× magnification of the scan.

### 2.7. Statistical Analysis

ANOVAS or Student’s *t*-test were used to determine *p*-values when comparing groups. Standard error or standard deviation was calculated to determine variation within a group. The inactivity index of each viral infection was calculated using the scores observed daily. For both the viral and antibody titers, specifically standard deviation was calculated within each experimental group.

## 3. Results

### 3.1. Study Design

**Figure 1:**
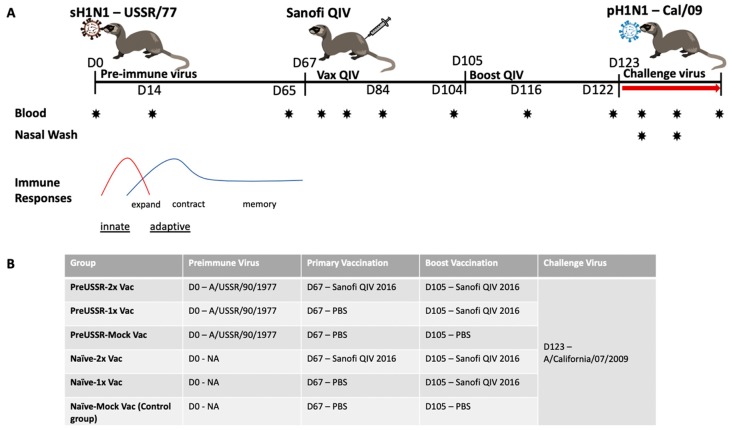
Study timeline and experimental group design for the investigation of influenza immune history and regulation of host responses to Sanofi FLUZONE^®^ QIV. (**A**) The study time shows the time course of infections and vaccinations in relation to the general dynamic trends of the innate and adaptive systems. (**B**) The Table depicts the experimental groups and corresponding preimmune virus, vaccine course, and challenge.

With the exception of neonates and young infants, the majority of the human population has been previously exposed to influenza viruses. By living through the winter influenza season or any time year-round in the tropics, people will be exposed to influenza viruses either from community transmission or vaccination. Considering this, understanding vaccine responses in the preimmune host will give a more accurate picture of the responses to vaccination for the majority of the human population. The early stages of influenza infection are defined by the antiviral response and innate immune cell activity [35]. Subsequently, adaptive cellular and humoral responses move through phases of expansion, contraction, and memory, to establish host long-term protection (Figure 1A) [24]. To understand how the preimmune host responds to vaccination, we designed a study using the ferret model to build an influenza preimmune background for subsequent vaccination and challenge studies. The design and experimental timeline of the study are shown in Figure 1A. The key to our study design was the elongated recovery period. Previous studies investigating the immune re-encounter with influenza viral antigens or live virus infection have frequently been designed with short recoveries of 14 to 28 days separating primary and secondary challenges [36,37,38]. These time frames do not properly account for the actual immunological recovery. Our rationale for this study was to allow the ferrets to recover over several months. Several studies have shown that influenza virus-specific immune cells are present in high numbers in circulation even 21 days after infection [22,23]. Our study design includes a long recovery period so that re-exposure (infection or vaccination) occurs after the contraction phase to more accurately reflect the seasonal exposures of influenza viruses in humans.

To establish preimmunity, adult ferrets were infected (imprinted) with a sublethal dose of the historical seasonal H1N1 A/USSR/90/1977 (USSR/77) on Day 0 of the study. After initial infection, we determined that the ferrets were imprinted if they were seronegative (determined by HAI assay) for circulating influenza virus strains and the imprinting virus prior to USSR/77 infection at Day 0 and had a USSR/77 HAI titre > 1:40 after infection. The USSR/77 imprinted ferrets are herein referred to as preimmune. USSR/77 was chosen as the imprinting virus since it was a previously circulating human influenza virus that would have significantly impacted the immune history of many people alive today [32]. Furthermore, USSR/77 is a virus that re-emerged in the 1970s and is antigenically divergent compared to our proposed contemporary challenge virus and the antigens present in the vaccine [13]. Ferrets recovered over 67 days to decrease non-specific immune responses. The ferrets were subsequently vaccinated intramuscularly in the hind limb on Day 67 post imprinting/infection (*p*i) and boosted on Day 105 *p*i with the Sanofi QIV split virion vaccine (FLUZONE^®^ Sanofi-Pasteur, PA, USA). To control the experiment, six groups (6 ferrets per group) were designed: (1.) preimmune USSR/77-QIV vaccinated and boosted (preUSSR-Vac2x); (2.) preimmune USSR/77-QIV vaccinated with no boost (preUSSR-Vac1x); (3.) preimmune USSR/77-mock vaccinated (preUSSR-Mock Vac); (4.) naïve-QIV vaccinated and boosted (Naïve-Vac2x); (5.) naïve-QIV vaccinated with no boost (Naïve-Vac1x); and (6.) naïve-mock vaccinated (Naïve-Mock Vac) (Figure 1B). To investigate protection from vaccination or preimmune infection, ferrets were infected with a currently circulating 2009 H1N1 pandemic virus on D123 *p*i. This virus, A/California/07/2009 (Cal/09), is one of the components of the Sanofi^®^ QIV vaccine (Sanofi Pasteur, North, York, Canada) used in our study. Furthermore, this strain is a representative from the 2009 H1N1 influenza pandemic which marked the emergence of an antigenically novel H1N1 “lineage” [39]. Since the vaccine contained Cal/09 antigen, vaccinated animals should be protected against challenge with Cal/09. Considering the USSR/77 infection and Cal/09 reinfection, this combination represents a monosubtypic heterologous reinfection. These viruses do not produce cross-reacting HAI antibodies when infected into the naïve ferret model, as we have previously shown [32]. The protein alignment in the highly variable region of HA (135-295aa), which belongs to the HA-RBD area, has only 59% homology between Cal/09 and USSR/77 [32]. This combination of preimmune exposure and challenge virus infection represents conditions plausible for the immune history of people alive today. Blood (serum), lungs, and nasal wash were collected at specified time points to examine the immune response. Specifically, blood samples were collected at Days 0, 14, 65, 68, 72, 79, 84, 104, 116, 122 *p*i and post challenge (*p*c) at Days 2 *p*c (125 *p*i), 7 *p*c (130 *p*i), and 14 *p*c (137 *p*i). These time points were chosen as they represented significant days surrounding the infection and vaccination events, as we have previously shown [28,34].

### 3.2. Milder Clinical Disease was Observed in the Preimmune-Vaccinated Ferrets at Challenge

Three groups of ferrets were infected with 1 × 10^6^ EID_50_ of the historical H1N1 strain USSR/77 at the start of the study and the temperature and weights were monitored in the ferrets over 14 days post imprinting (*p*i) (Figure S1). The remaining ferrets in other groups were left in control rooms to be age-matched for subsequent time points in the study. Analysis of the temperature and weight change of each group indicated minimal variation between groups following imprinting infection. All groups lost a moderate amount of weight following infection. At nadir, all groups had lost between 5% and 10% of their original weight similar to our previous findings with this virus [32]. All groups had a temperature increase peaking Day 2 *p*i. On Day 67 and Day 105, the ferrets were vaccinated and boosted, respectively, as specified by their group (Figure S2). Minimal weight loss and temperature change was noted over the 14-day period post vaccination and boost as we monitored for reactogenicity.

**Figure 2:**
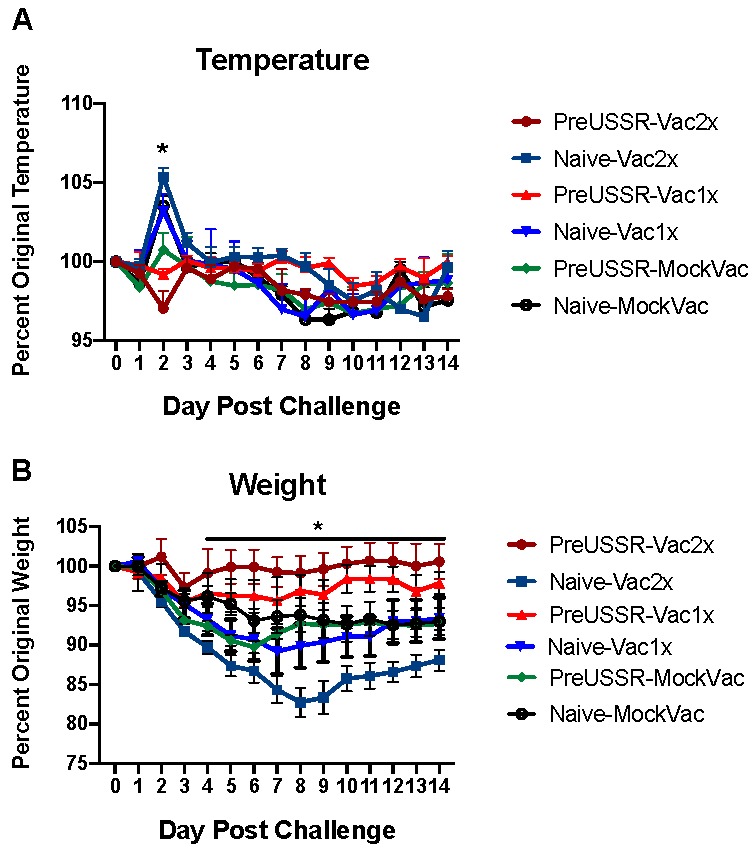
Previous imprinting with a historical H1N1 influenza virus increases influenza vaccine protection. Ferrets were infected with the historical H1N1 virus USSR/77 (10^6^ EID_50_) to establish an influenza-specific immune background or were left naïve. After 67 and 105 days, the vaccine group were vaccinated with the Sanofi FLUZONE^®^ QIV vaccine. All groups were challenged at Day 123 with the contemporary Cal/07 H1N1 pandemic 2009 virus. (**A**) Temperature and (**B**) weight changes were monitored for 14 days *p*c. * indicates a *p*-value of less than 0.05.

To determine how well the vaccine was able to protect against disease at challenge, on Day 123, the ferrets were challenged intranasally with Cal/09 at an infectious dose of 10^6^ EID_50_. The ferrets were monitored for signs of clinical disease including weight loss, fever, and lethargy post challenge. The preUSSR-Vac2x did not have a temperature increase *p*c, but instead experienced a slight drop in body temperature (Figure 2A). Furthermore, the preUSSR-Vac2x group lost minimal weight. Maximum weight loss was observed in this group on Day 2 *p*c where weight dropped to 97% of original weight (Figure 2B). Mild disease was also experienced by the preUSSR-Vac1x group although the weight loss period for this group was longer than that seen in the preUSSR-Vac2x group and maximum weight loss was noted at 96% of original weight. No temperature increase was observed in the preUSSR-Vac1x group. Conversely, the naïve-Vac2x and naïve-Vac-1x groups both experienced significant temperature increase to 105% and 103% of original temperature at Day 2 *p*c, respectively (*p* < 0.05 by *t*-test compared to preUSSR-Vac2x). These groups, as well as the control group (naïve-MockVac), had significant weight loss compared to the preUSSR-Vac2x group. Interestingly, the naïve-Vac2x group had the largest amount of weight loss pc where maximum weight loss was seen on Day 9 *p*c at 82% of original weight. Together, these results suggest that preimmune ferrets had protection following vaccination as shown by a milder clinical disease.

### 3.3. Decreased Respiratory Infection in Preimmune-Vaccinated Ferrets

**Figure 3:**
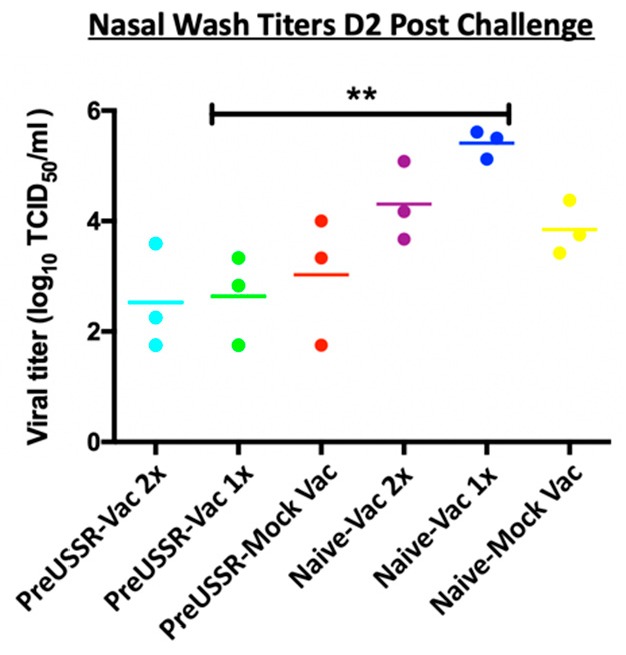
Viral shedding following challenge was lower in previously infected and vaccinated ferrets. Nasal washes were collected from all groups Day 2 *p*c (Cal/09). Live viral load was calculated by TCID_50_ titration assay using MDCK cells. Student’s *t*-tests were performed among groups to determine statistical significance. ** indicates a *p*-value of less than 0.001.

Hallmarks of influenza virus-induced illness include respiratory virus shedding and lung pathology. To evaluate susceptibility to viral infection and respiratory disease, we collected nasal washes (NW) and lungs from ferrets pc with the Cal/09 H1N1 pandemic virus. NW were collected Days 2 and 7 *p*c. Virus was only detected in NW collected on Day 2 *p*c and results of viral titering are shown in Figure 3. Titers in the preUSSR-vaccinated groups were markedly or significantly lower than the naïve-vaccinated or naïve-unvaccinated control groups. Both preUSSR-vaccinated groups had viral titers averaging at 2.5 log_10_ TCID_50_. Furthermore, the naïve-Vac1x group had the highest titer of viral shedding (Figure 3).

**Figure 4:**
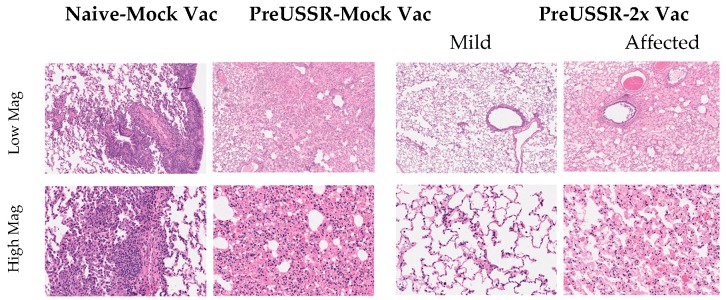
Histopathological analysis shows differential lung pathology on Day 14 post challenge dependent on influenza virus and vaccination history. Respiratory tissue was collected from ferrets Day 14 post challenge and the lungs were processed for histopathological assessment. Tissue morphology was assessed by hematoxylin & eosin staining. Lungs were analyzed by microscopy from at least three ferrets per group. Images were captured as described and representative images from the groups Naïve-Mock Vac; PreUSSR-Mock Vac; and PreUSSR-2x Vac are shown. High resolution scans were performed using an Aperio ScanScope XT (Leica Biosystems, Concord, Canada) at 40× magnification. Images of the scans were captured using the HALO program from UHN AOMF (Advanced Optical Microscopy Facility, Toronto, Canada) at 5× (low) or 20× (high) magnification of the scan.

Histopathological analysis of the lungs collected on Day 14 *p*c with Cal/09 suggested distinct immune responses in the respiratory tract which were dictated by influenza immune background. Lungs were collected at necropsy and prepared for histopathological analysis (H&E staining). Stained and mounted lung sections were viewed at High (20×) and Low (5×) magnification (Figure 4). The Naïve-MockVac group showed typical Day 14 pathological features of Cal/09 infection including mononuclear cell infiltration, bronchiolar wall thickening, and hemorrhage (left panels) [31]. The preUSSR-MockVac lungs had evidence of necrotising alveolitis (middle left panels). The preUSSR-Vac2x group ferret lungs had evidence of either mild pathological changes characterized by minimal leukocyte infiltration, peribronchiolar thickening, and alveolar wall thickening (mild) or significant pathological features illustrated by interstitial pneumonia with alveolar wall thickening, edema, and fibrosis (affected) (right panels). Taken together, the analysis of viral load shedding and histopathology suggested that the clinical outcomes in the respiratory tract after Ca/09 challenge were dependent on the immune background in regard to the permutation of vaccination and previous infection.

### 3.4. Humoral Immunity is Differentially Regulated in the Preimmune Host

**Figure 5:**
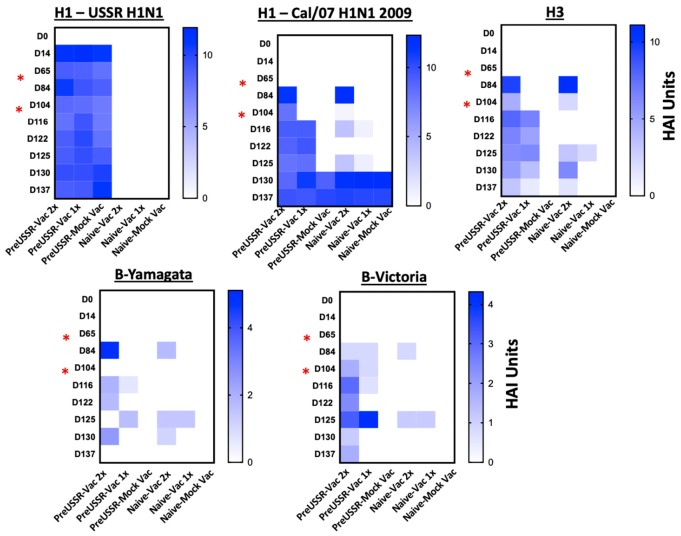
Increased antibody titers directed toward vaccine antigens in previously infected ferrets. Haemagglutination inhibition assays were performed using serum collected from ferrets from each group throughout the time course. The reciprocal of the largest dilution capable of inhibiting red blood cell agglutination was determined to be the HAI titer. To calculate the HAI Units, a log2 was taken from the dilution factor. The mean HAI Units were calculated for each experimental group and the results were plotted in heat maps to visually represent changes in antibody titer and specificity over time. HAI assays were performed using specific whole virus (USSR/77) or BPL inactivated vaccine antigens with turkey red blood cells. Previously infected ferrets elicited greater antibody titer which were longer lived in circulation compared to naïve-vaccinated ferrets. Red asterisks indicate vaccination days.

**Figure 6:**
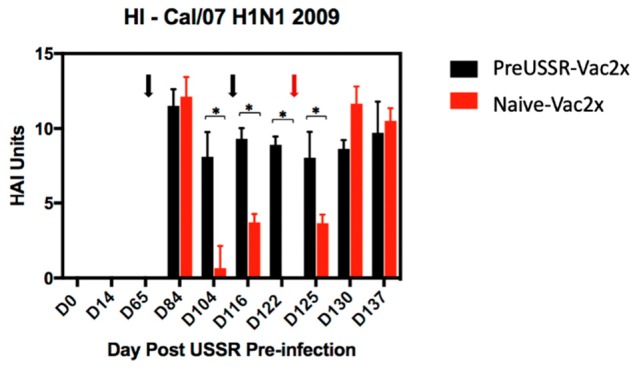
Focused titer analysis of the H1-2009 specific antibodies elicited in the PreUSSR-Vac2x and Naïve-Vac2x groups. HAI assay utilizing serum collected throughout the time course for the preUSSR-Vac2x and Naïve-Vac2x groups against the 2009 H1N1 pandemic Cal/09 virus are graphed to better visualize quantitative and statistical differences based on the contribution of preimmunity. Black arrows denote time of vaccination. Red arrow denotes the challenge day. Day 0 is the time of infection with the seasonal H1N1 USSR/77 virus. Values are graphed in HAI Units. * indicates a *p*-value of less than 0.05 as determined by Student’s *t*-test.

HAI assays titering serum antibodies specific to influenza virus HA proteins are considered to be the standard correlate of immunity predicting clinical outcome following an influenza virus infection. To determine the antibody dynamics relative to clinical outcome, we next investigated the HA specific antibody responses in each group over the experimental time course. Sera collected from the experimental groups along the experimental timeline (Days 0, 14, 65, 84, 104, 122, 125, 130, 137) were analyzed. Standard HAI assays were performed to quantify antibody specificity and regulation against influenza HA antigens (Figure 5 and Figure 6). USSR/77 virus was used to quantify antibodies reactive to this antigen, whereas the 2016 WHO kit was used to quantify antibodies toward the vaccine antigens: 2009 H1, H3, B-Yamagata, and B-Victoria antigens.

Heat maps of the calculated HAI units (Log2 of the reciprocal HAI titer) averages for each group at each time point were generated to visually summarize the HAI data with respect to seasonal USSR/77 H1, pandemic Cal/09 H1, H3, B-Yamagata, and B-Victoria HA antigens over the 137-day time course (Figure 5). HAI units for each sample time point are also depicted to give a more detailed view of the changes in elicited antibodies over time (Figure S3). The mean HAI units per group per time point was calculated over the entire study. The H1-USSR heat map (top left graph) shows strong HA antibody generation by Day 14 following infection for all infected groups. All ferrets infected with USSR/77 (H1-USSR) developed HA binding antibodies to the H1 USSR/77 antigen between 10 and 12 HAI Units as assessed on Day 14 *p*i. Titers in these previously infected animals remained between 7 and 12 HAI Units for the entire study. The uninfected ferrets remained negative throughout the study. At vaccination and boost (Day 67 and Day 105, respectively), the USSR/77 infected ferrets had strong HA antibody responses shown by high HAI titers to the H1 2009 HA protein (top middle graph). These H1 Cal/09 antibody titers elicited following vaccination in the preUSSR group remained high until the end of the study. Following first vaccination, both previously infected and naïve ferrets developed high antibody titers to the H1 2009 HA (H1-2009), between 10 and 14 HAI Units, shortly after vaccination (Day 14). When titers were quantified in sera collected at a longer time points (30 days) following vaccination, the preimmune ferrets had sustained antibody values between 6 and 10 HAI Units whereas 4/5 of the naïve-vaccinated ferrets had undetectable levels of H1 Cal/09 reactive antibodies. Fourteen days following boost, the preUSSR-Vac2x ferrets had H1 Cal/09 titers between 8 and 10 compared to lower values seen in the naïve-Vac2x ferrets (between 3 and 4 HAI Units). Furthermore, H1 Cal/09 antibody titers assessed prior to vaccination showed no detectable levels and between 8 and 11 for the naïve-Vac2x and preUSSR-Vac2x ferrets, respectively, suggesting no cross-reactivity detected by HAI for antigens Cal/09 and USSR/77. The naïve-Vac1x ferrets also had strong or moderate responses to the vaccine directly following vaccination but these titers quickly waned and were not maintained over time. Interestingly, these trends were consistent for the other HA antigens H3, B-Yamagata, and B-Victoria, where the preUSSR ferrets had a stronger antibody response following vaccination and boost despite the antigenic divergence of the vaccine antigens with the original imprinting virus. Taken together, these results suggest that a previous infection with an influenza virus primes the naïve immune system to have greater antibody responses following vaccination with the Sanofi Fluzone QIV^®^ (Sanofi Pasteur, North York, Canada) vaccine administered by intramuscular injection despite divergence in antigenicity.

To visualize numerical changes in antibody titers across the experiment for the H1 Cal/09 antigen, HAI values were determined for each ferret and the group averages were calculated per time point and plotted in a histogram. For simplicity, only the results for PreUSSR-Vac2x and Naïve-Vac2x are shown (Figure 6). Serum collected on Day 84 following first vaccination showed similar HAI levels between the two groups (>11 HAI Units). Interestingly, the subsequent blood collection prior to boost indicated a significant drop in Cal/09 H1 antibody titer in the naïve-Vac2x groups to <2 HAI Units whereas the PreUSSR-Vac2x group had a minimal drop in HAI to 8 HAI Units. Statistical differences in HAI titers for the Cal/09 H1 antigen between the preUSSR-Vac2x group and the naïve-Vac2x group were seen throughout the time course until Day 7 *p*c.

### 3.5. Mature B Cells Play a Role in the Vaccine Responses of the Imprinted Host

**Figure 7:**
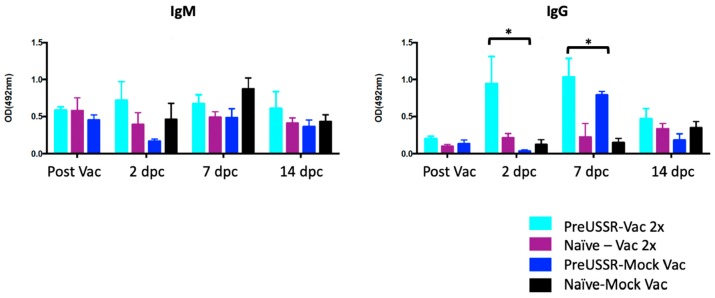
Increased virus-specific IgG isotype serum antibody titers following challenge in the preimmune-vaccinated ferrets compared to naïve-vaccinated ferrets. Isotype ELISAs were performed using sera collected from each group throughout the time course to quantify the circulating virus-specific IgG and IgM antibodies. Only time points post vaccination and challenge are shown Post Vaccination, Day 2 *p*c, Day 7 *p*c, and Day 14 *p*c. A Student’s *t*-test was conducted to compare with results on Day 0, * *p* < 0.05.

Above, we found that the ferrets previously exposed to an antigenically divergent influenza virus had stronger antibody responses to Sanofi QIV^®^ vaccination. From these findings, we next investigated the dynamics of the antibody isotype produced during the sequential infections and vaccinations of our study. To do this, we performed virus-specific IgG and IgM ELISAs to quantify the isotype specific for the Cal/09 virus. Sera collected from all time points were evaluated but only the time points prior to and following challenge (D2, D7, and D14) are shown (Figure 7). No statistical differences were found in the IgM levels among each group throughout the time course with the exception of the naïve-MockVac group, which did not have any virus-specific IgM antibodies until after challenge (left graph) as expected. All IgM titers remained oscillating around 0.5 OD 492 nm. Quantification of virus-specific IgG production found statistical differences in IgG levels among the groups Day 2 and Day 7 *p*c (right graph). Specifically, the preUSSR-Vac2x group had statistically higher IgG levels (OD = ~1) than all other groups, which had lower values closer to 0. The Naïve-MockVac group was negative for IgG antibody production until after challenge as expected. The preUSSR-MockVac group had significant increases in IgG on Day 7 following infection. Comparing IgM and IgG levels, we did not observe a trend of higher IgG compared to IgM overall.

**Figure 8:**
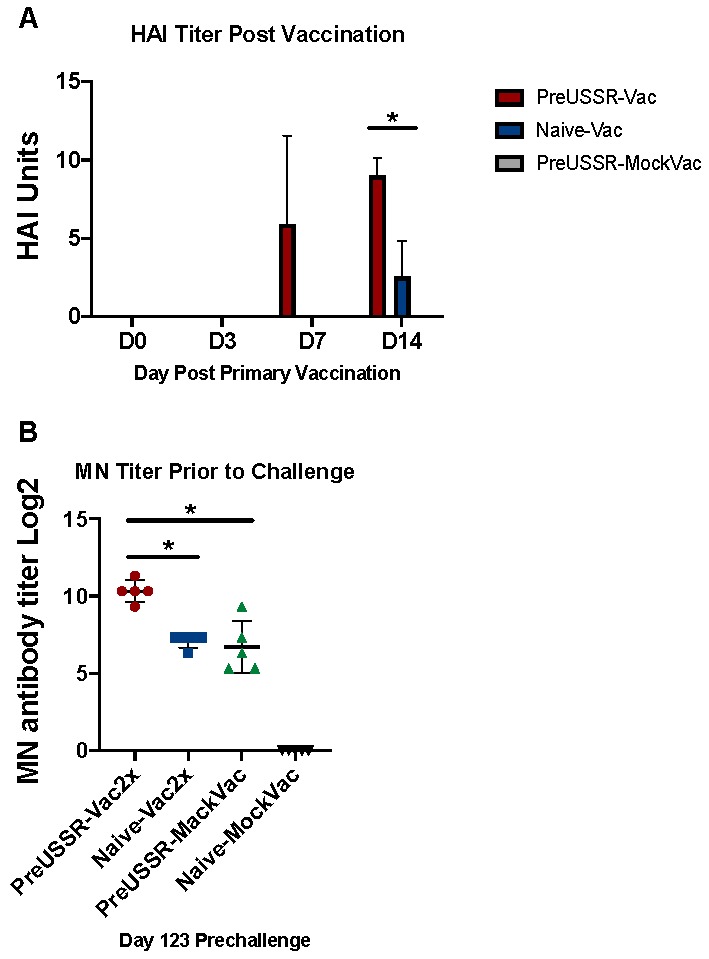
Antibodies elicited post vaccination in the PreUSSR-Vac2x ferrets were detected earlier and had increased neutralization activity compared to other experimental groups. Serum was collected at early time points after primary vaccination (Days 0, 3, 7, and 14 post vaccination). (**A**) HAI assays were performed as previously described using sera isolated from blood collection post vaccination against the Cal/07 (pH1N1) virus to determine the dynamics of antibody generation after vaccination. (**B**) Microneutralization assays were performed by standard protocol on MDCK cells using serum from all experimental groups post vaccination and prior to challenge. For simplicity, only the PreUSSR-Vac2x, PreUSSR-MockVac, Naïve-Vac2x and Navie-Mock Vac groups are shown. Student’s *t*-test was conducted to compare results between each group, * *p* < 0.05.

Since the preimmune-vaccinated ferrets had an increase in IgG isotype compared to other groups, this suggested that the preimmune-vaccinated groups had an increase in previously established B cell responses due to the imprinting event. To better understand the immediate responses following vaccination and investigate if this was a recall or new response, we evaluated the antibody dynamics shortly after vaccination in the preimmune-vaccinated and naïve-vaccinated groups. We collected blood in the early time points following vaccination (Days 0, 3, 7, and 14) from naïve ferrets, ferrets previously infected with USSR/77, and naïve-mock vaccinated ferrets as control (Figure 8A). HAI assays were performed with the serum against the Cal/09 H1 antigen and showed that all groups were negative or had undetectable levels of antibody reactive toward Cal/09 H1 at Days 0 and 3. By Day 7 the preUSSR-Vac group had a positive HAI response to Cal/09 H1, reporting at ~5 HAI Units (light blue bars), whereas both the vaccinated alone and mock vaccinated group did not have a positive HAI. Furthermore, by Day 14, the preUSSR-Vac group had a strong HAI response at 9 HAI Units compared to the naïve-vaccinated ferrets which had a mean of 3 HAI Units for the group (light purple bars). These results suggest that the ferrets with a previous but antigenically divergent H1N1 virus infection generated a faster and stronger response to the new 2009 H1 antigen, possibly due to a pre-exisiting clone.

To investigate the possible mechanism leading to greater protection in the preimmune-vaccinated ferrets, we next investigated the function of the antibodies produced over the time course by performing microneutralization (MN) assays (Figure 8B). Using serum isolated from blood collected pre-challenge, MN assays were performed as previously described against the Cal/09 virus [28]. Pre-challenge serum (Day 123) was used for the MN assays as an indicator or predictor of clinical disease observed during challenge. The preUSSR-Vac2x group had the highest titer of MN antibodies at ~10, which was statistically greater than any other experimental group. Furthermore, both the preUSSR-MockVac and the naïve-Vac2x had lower MN titers compared to the preUSSR-Vac2 group but similar MN titers to each other. As expected, there were not any MN titers detected for the naïve-mock group. Together, these data show that the preUSSR-Vac2x group had the highest titer of functional antibodies capable of inhibiting viral infection.

**Figure 9:**
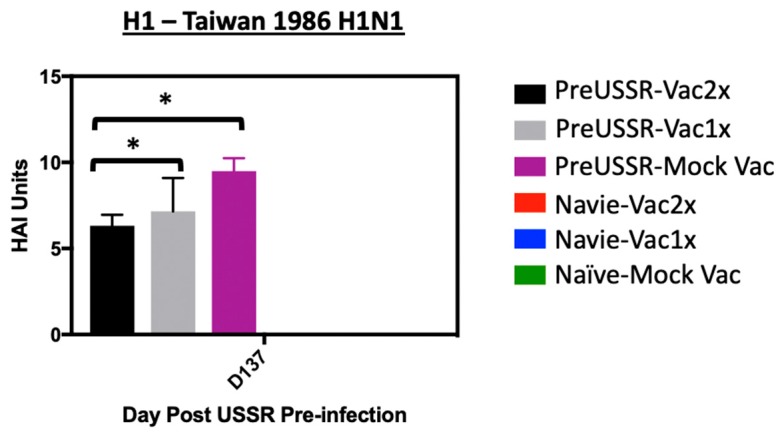
Decrease in cross-reactive antibody titer over sequential influenza virus exposures of antigenically divergent strains. Serum was isolated from blood collected at end day (Day 137) from all ferrets. Serum was then used for HAI assays against an antigenically divergent influenza virus strain (A/Taiwan/1/1986, Taiwan/86) as a read-out for the production of cross-reactive antibodies. Standard HAI assays were conducted with turkey RBCs against Taiwan/86. Student’s *t*-test was conducted to compare results with the PreUSSR-Mock Vac group, * *p* < 0.05.

Previously, sequential infections with divergent influenza viruses have been shown to elicit antibodies that are more broadly reactive toward a larger spectrum of antigenically distinct influenza strains [15,40,41]. Although these studies showed the induction of broadly neutralizing antibodies to be through an HA stem mechanism, we were interested in knowing whether our sequential exposures, historical H1N1 virus infection → vaccination → challenge, had an effect on the specificity and cross-reactivity of the antibodies elicited over time. Therefore, we performed additional HAIs using the antigenically distinct influenza virus strain A/Taiwan/1/1986 (Taiwan/86) with our serum samples. Taiwan/86 is antigenically removed from both USSR/77 and Cal/09 and has 90% homology to USSR/77 and 62% identity to Cal/09 as analyzed in the HA receptor binding domain area (a.a. 135–295) [32]. Previously we showed that direct infection with Taiwan/86 did not elicit cross-reactive antibodies toward either USSR/77 or Cal/09 viruses [32]. In this study, we also showed that the reverse was also true, infection with USSR/77 or Cal/09 did not elicit cross-reactive antibodies toward Taiwan/86 as determined by HAI assays. These results suggest that any cross-reactivity observed our sequential infection study would be elicited due repeated influenza antigen exposure. A HAI assay was performed using serum isolated from collection on Day 14 *p*c (Day 137 of the entire study) since this serum would be representative of the largest number of sequential exposures: PreUSSR→QIV→QIVBoost→Challenge. The assay was performed against the Taiwan/86 virus and the results showed distinct trends in HAI cross-reactivity related to the multiplicity of exposures. Specifically, the preUSSR-MockVac ferrets had the highest titer of cross-reactive antibodies against Taiwan/86 observed at 9 HAI Units (Figure 9). Both vaccinated groups had successively lower HAI titers with an inverse relationship to the number of repeated exposures. Specifically, the preUSSR-Vac1x and preUSSR-Vac2x had average group titers of 7 and 6 HAI Units, respectively. This data suggests that a low number of sequential exposures could elicit a high titer of cross-reacting antibodies to a highly divergent strain but the more influenza exposures that occur afterward refined the response.

## 4. Discussion

Each year, the influenza vaccine has variable effectiveness against seasonal circulating viruses allowing continual virus circulation in humans. Due to continual circulation facilitating constant influenza virus exposure, humans have a complicated influenza background. The split virion vaccine is most often used for seasonal influenza vaccination worldwide, but there has been limited analytical and experimental investigation of vaccine protection in animal models imprinted with historical or contemporary human influenza viruses. To investigate the imprinted host-responses to the Sanofi QIV^®^ vaccine, we developed a ferret model on a USSR/77 influenza virus background for investigating the antibody responses during vaccination and challenge. Our model had a prolonged recovery period in an attempt to better replicate the lag time and subsequent immune maturation between sequential influenza antigen exposures. We found that the imprinted ferrets had increased antibody responses to vaccination which led to increased protection at challenge. Together, our results suggest that much could be learned from the immune responses to vaccination in the preimmune host and that the mechanisms identified could be leveraged for improving vaccines.

As mentioned above, the objective of our study was to understand how previous infection with a historical H1N1 influenza virus influences QIV vaccination and contemporary H1N1 viral challenge. Our results suggest that previously infected ferrets are better immunologically equipped to respond to the split virion influenza vaccine. We showed that the ferrets preimmune to the USSR/77 virus were able to mount a greater and longer sustained antibody response as determined by HAI assay toward all vaccine antigens. Furthermore, the antibodies elicited had greater neutralization activity suggesting that the mechanism of milder disease at challenge was the ability to develop more functional virus inhibiting antibodies. Similar trends showing increased inactivated influenza virus vaccine responses in primed Live Attenuated Influenza Vaccine (LAIV) vaccinated ferrets have been reported suggesting that a conserved priming effect may occur after a live virus exposure [42,43,44,45]. Using ferret and African Green Monkey models as well as human subjects, these studies showed that priming effects were seen after LAIV administration in the respiratory route but not from priming with inactivated virus vaccination through the intramuscular route [44] indicating the importance of site-specific respiratory tract priming prior to inactivated vaccination. Specifically, in a study that investigated potential pandemic H7 influenza vaccines, priming human subjects with a pandemic LAIV H7 vaccine was able to induce a greater antibody response when the volunteers were vaccinated later with an inactivated H7 formulation [45]. Similarly, in a study that used African Green Monkeys to investigate LAIV priming prior to inactivated vaccine administration for H5 viruses, Jegaskanda and colleagues found that LAIV priming led to memory B cell responses localized to the mediastinal lymph node which was subsequently boosted with intramuscular administration of an inactivated virus vaccine by stimulating germinal center reactions in peripheral lymphoid tissues including spleen and other lymph nodes. Sequence analysis of the immunoglobulin heavy and light chains indicated that VH3 and VH4 were the typical gene families induced after the prime-boost regime giving further support of active germinal center (GC) reactions with peripheral expansion outside of the mediastinal lymph node. These findings were similar to our results with regard to subsequent intramuscular administration of the split-virion influenza vaccine which induced a more robust peripheral response that was not seen in the naïve or unprimed ferrets. Future investigation of site-specific memory B cell and plasmablast responses in the draining lymph nodes of the respiratory tract and the peripheral immune sites, such as the spleen, bone marrow, and inguinal lymph nodes, will give more insight into the mechanism of the protection we observed in the imprinted-vaccinated ferrets. It is important to note that the LAIV vaccines used in these previous studies were designed to only replicate in the upper respiratory tracts while imprinting using the historical H1N1 has a greater tropism in the respiratory tract which may affect the immune mechanisms of protection. Furthermore, the inactivated vaccine used in our study was quadrivalent, while the vaccines used in these previous studies were monovalent. It would be interesting to identify whether immune specificity is influenced by additional antigens at vaccination.

In our study, we also found that the naïve-vaccinated ferrets developed a more severe disease compared to other groups, which may suggest that split virion vaccination on a naïve background may lead to the development of antibodies that are not as efficient at inhibiting viral infection. We hypothesize that vaccine protection as studied here in naïve-vaccinated mature animals has not been fully explored in humans since the majority of humans are preimmune to influenza viruses prior to vaccination. Infant humans would be the major accessible naïve population to investigate vaccine responses but are immunologically immature and not directly comparable to our control group. Although we recognize this limitation in our study with regard to increased disease in the naïve-vaccinated ferrets, we do draw similarities between the immune responses elicited in influenza virus naïve infants and our naïve ferrets after vaccination. Infants have decreased antibodies elicited at influenza vaccination that wane faster than preimmune adults which is a similar trend in our naïve-vaccinated ferrets [9,46,47]. Understanding how a prior infection primes the immune system for greater protection at vaccination may offer new strategies for priming the immune system in the naïve hosts which our model attempts to represent. More work is needed to elucidate the molecular mechanisms of eliciting neutralizating antibodies for the development of more effective vaccine platforms.

During influenza virus infection, a large and diverse pool of antibodies are initially produced and circulated [18]. The majority of these are directed toward the HA molecule [48,49]. The initial humoral response to infection involves the generation of short-lived plasma cells (low-affinity antibody producing cells) that reside in secondary lymphoid organs [21]. These cells expand rapidly, then decline prior to memory establishment. In the germinal centre, B cell specificity is refined for improved responses toward the pathogen. Refinement occurs through a process called affinity maturation. Affinity maturation includes somatic hypermutation (SHM), class-switch recombination (CSR), and clonal selection [50]. Our results from antibody isotype ELISAs and early time point HAIs showed that preimmune ferrets had a quicker development of vaccine antigen specific antibodies following vaccination and increased IgG isotype compared to the other groups. The increase in virus-specific IgG in the preimmune-vaccinated group compared to other groups suggested the presence of a pre-existing B cell with either existing vaccine antigen specificity or the presence of a pre-existing B cell with the flexibility to modify antigen specificity. Further investigation of affinity maturation during sequential exposure of antigenically diverse exposures should be approached to understand how much of a role affinity maturation plays in continual influenza virus infection and vaccination. Moreover, we also found a trend of decreased cross-reactive antibody production over the sequential influenza viral antigen exposure. The ferrets that had the most viral antigen exposure events—imprinting, primary vaccination, vaccination boost, and challenge—had the lowest HAI titer against the antigenically divergent A/Taiwan/1/1986. This was an interesting result as it showed that there were shifts in the antibody reactivity pool over multiple viral exposures and suggested that the breadth of the response was decreased. It is important to note that this HAI analysis did not indicate specific antibody affinity or epitope dominance. Future investigations should also include defining the affinity and epitope dominance or retention of the elicited antibodies over experimental sequential viral antigen exposures.

Local inflammatory and immune memory generation in the respiratory tissue define the primary immune response which impacts future exposures. Tissue resident memory T cells are known to be induced and retained in the lungs following respiratory virus infection [51]. Recent evidence has suggested that there is also a specific respiratory memory B cell response retained in the lungs of influenza virus infected animals [52]. These memory B cells have a specific phenotype and function which is distinct from circulating memory B cells. These cells are rapidly stimulated following a secondary challenge for the induction of plasmablasts. This new evidence presents questions regarding activation of the respiratory memory B cells at long-distance stimulation such as by the intramuscular vaccinations. It is of interest to investigate if respiratory memory B cells are activated following split-virion influenza vaccination by intramuscular injection despite the large distance from the respiratory tract. Additionally, viral antigen retention either through persistence of viral proteins or nucleic acids may also have a role influencing the outcomes in our preimmune-vaccination study. Viral antigen persistence has been documented in the mouse model, where after clearance of the virus following a primary nonlethal infection, depots of viral antigen may exist for long periods of time that regulate memory T and B cell responses. Dendritic cells can retain viral antigen for presentation to the T cell in the draining lymph nodes to allow continual pathogen specific immune responses. The literature suggests that viral antigen persistence may last up to 30 days post influenza virus infection in the mouse model [53]. Investigation of this phenomenon in our ferret preimmune-vaccination model would be of interest to determine if viral persistence is playing a role in skewing immune responses at the vaccination event. Furthermore, noting the length of viral persistence would be important when considering our designed recovery period.

Outside of B- and T-cell-specific responses, there are other immune mechanisms that may be responsible for the faster generation of antibodies following vaccination in the imprinted host as we observed. Specifically, trained immunity is the adaptation of innate immune components after pathogen stimulation. Trained immunity mainly involves the modification of cells such as NK cells, monocytes, and macrophages to produce a “memory” response which can be mobilized over a longer period of time for a second encounter with a specific pathogen [54]. It is possible that either trained immunity or memory B cell dependent responses influenced the specificity of the elicited antibodies after vaccination in the imprinted hosts. However, considering our long rest period between infection and vaccination, trained immunity is less likely. Further investigation of B cell memory and refinement compared to trained immunity will be important to determine the immune mechanisms. Understanding the roles of these in the imprinted host’s responses to vaccination may be critical for developing the next generation of influenza vaccines.

Due to the nature of the drifting and shifting influenza viruses, there has been much work investigating immune responses in infection-reinfection studies using animal models. Since the emergence of the pandemic 2009 influenza virus, there have been several experimental and human investigations on monosubtypic heterologous infection-reinfection due to the occurrence of this infection sequence in the human population. The general finding from these studies was that primary infection with seasonal H1N1 from the 1977 H1N1 lineage followed by infection with pandemic 2009 virus leads to partial protection and reduced clinical severity in infected individuals [55,56,57,58,59,60]. We previously reported that primary infection induced pre-existing non-HA antibodies during 2009 H1N1 virus challenge due to sequence homology of less immunogenic internal viral proteins. Other reports showed this sequential infection leads to the generation of both HA head and stalk antibodies and that stalk antibodies may be responsible for a more broadly protective antibody response [15,40,41,61]. Together, these reports suggest that antibodies generated toward internal proteins and/or the HA stem may also play a role in the clinical outcome we observed. Although we can glean information on the immune responses in the pre-exposed hosts from the experimental infection-reinfection studies, it is important to note that our study involved sequential viral antigen exposure both in the forms of live viral infection and inactivated viral antigens at vaccination. Our study specifically showed a broader antibody response in the preimmune ferrets directed toward the HA head as shown in our HAI assays which was not recognized in these other studies. Future investigation using a live virus sequential infection study similar to previous studies [40,41] but including a long recovery periods should also be approached. Furthermore, the limitation of our study is that we only investigated antibody specificity by HAI assays. Antibodies elicited toward the HA stem and other viral proteins including the neuraminidase protein in a long recovery model should also be done to gain a more complete picture of the humoral immune responses in the preimmune host during vaccination.

Although great efforts have been made to develop more protective influenza virus vaccines, there has been little progress improving vaccine effectiveness as evidenced by the moderate overall effectiveness of the 2018–2019 vaccine at 47% [62]. Several vaccine formulations now exist but it is not known how the preimmune host will response to these platforms. The Centers for Disease Control and Prevention estimated over 80,000 deaths occurred due to influenza virus infection in the 2017–2018 influenza season. Our study takes a step forward to understanding the actual vaccine responses in the majority of humans, since all people over the age of 4 will have an influenza immune history [13].

## 5. Conclusions

Together, our findings suggest that H1N1 influenza immune priming leads to greater protection at vaccination. Determining how to prime the immune system without the deleterious effects of infection should now be an essential research goal. Importantly, our results may only be extrapolated to human situations with a single previous infection prior to vaccination. Using our strategy, further research is needed to tease out the protection and vaccine responses on alternate influenza virus backgrounds such as H3N2 or a layered serial influenza background [63]. In addition, preimmune host responses to vaccination with other vaccine platforms, such as the Live Attenuated Influenza Virus (LAIV) vaccine, should also be investigated.

## Supplementary Materials

The following are available online at https://www.mdpi.com/2076-393X/7/4/133/s1. Figure S1: Weight loss and temperature change following infection with A/USSR/90/1977 to establish a preimmune background. Figure S2: Histogram visualization of HAI titer regulation over the experimental time course found preimmune ferrets had greater antibody responses compared to naïve-vaccinated ferrets. Figure S3: Minimal weight loss and temperature change were observed following vaccination with Sanofi FLUZONE^®^ QIV.
