# Supplementary material for: Historical H1N1 Influenza Virus Imprinting Increases Vaccine Protection by Influencing the Activity and Sustained Production of Antibodies Elicited at Vaccination in Ferrets
Source: Vaccines (Basel). 2019 Sep 28;7(4):133. doi: 10.3390/vaccines7040133 (PMC6963198; doi:10.3390/vaccines7040133)
Supplement: Supplementary file 1 [file vaccines-07-00133-s001.pdf]

Supplementary Materials

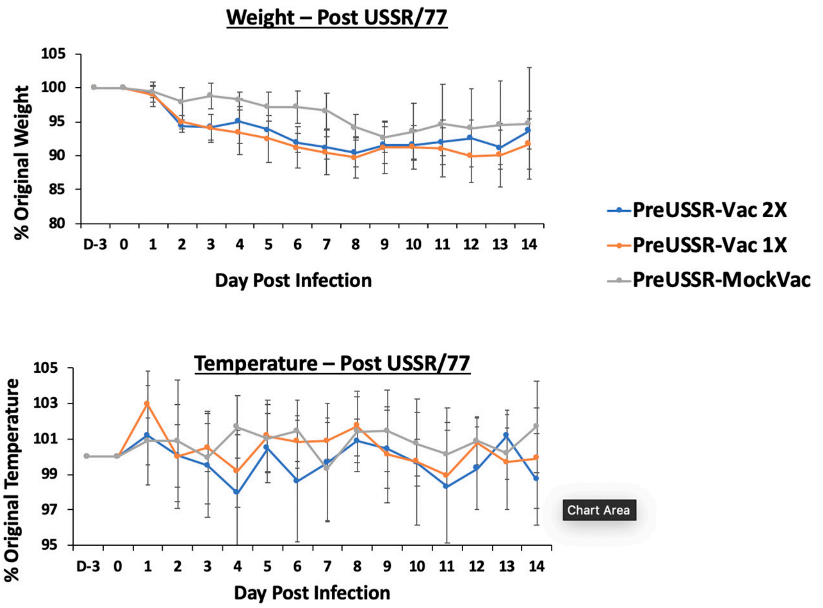

Figure S1: Weight loss and temperature change following infection with A/USSR/90/1977 to establish a preimmune background. Ferrets randomly distributed into groups and then intranasally inoculated with A/USSR/90/1977 in PBS at a dose of 10^6 EID_{50}. Ferrets were weighed and temperature was observed daily. The percentage of original values was calculated throughout 14 days.

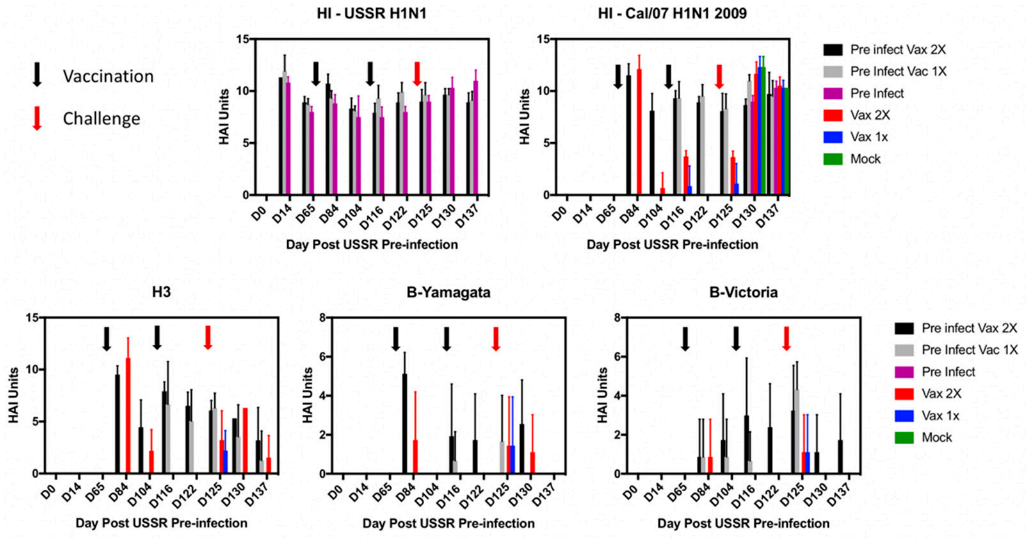

Figure S2: Histogram visualization of HAI titer regulation over the experimental time course found preimmune ferrets had greater antibody responses compared to naïve-vaccinated ferrets. The HAI U for each experimental group was calculated at each time point for the HAI titer against the Cal/09 virus. The results were plotted as histograms over the infection time course to visualize the numerical

dynamics over time. HAI values are plotted per antigen/virus. Time of vaccination is indicated by a black arrow. Time of challenge is indicated by a red arrow. Day 0 denotes the first day of the study (H1N1 USSR/77 virus inoculation day in appropriate groups).

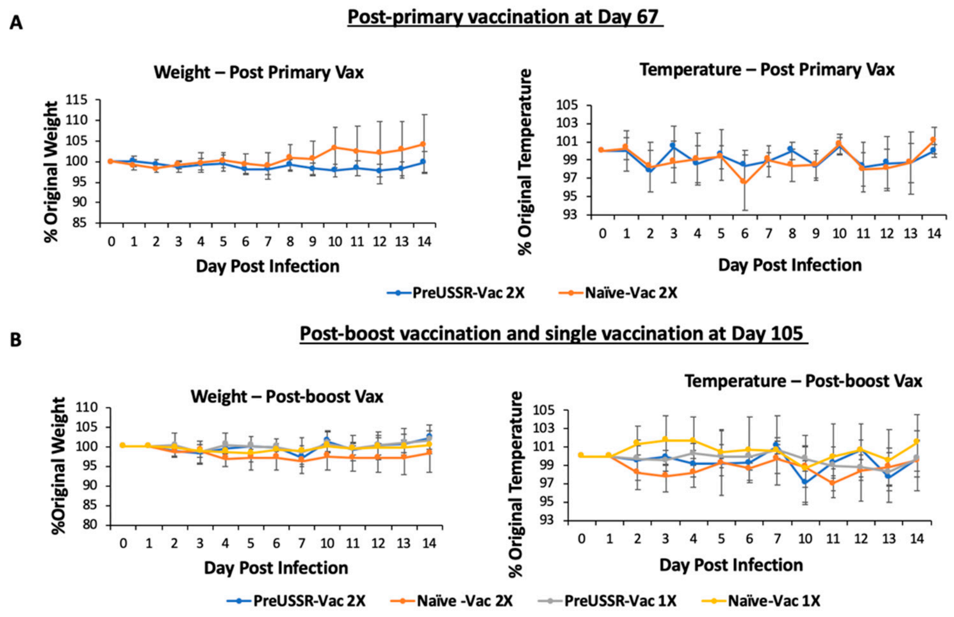

Figure S3. Minimal weight loss and temperature change were observed following vaccination with Sanofi FLUZONE® QIV. Both Naïve and preimmune ferrets were vaccinated by intramuscular injection with the Sanofi FLUZONE® QIV split virion vaccine on Day 67 and/or Day 105 following the initial imprinting infection. Ferrets were weighed and temperature was recorded daily. The percentage of original values was calculated and graphed throughout 14 days.
